# A comparative study of the effectiveness and safety of combined procarbazine, lomustine, and vincristine as a therapeutic method for recurrent high-grade glioma: A protocol for systematic review and meta-analysis

**DOI:** 10.1097/MD.0000000000022238

**Published:** 2020-09-18

**Authors:** Yang Cai, Yu-Gang Jiang, Ming Wang, Zhuo-Hang Jiang, Zhi-Gang Tan

**Affiliations:** Department of Neurosurgery, The Second Xiangya Hospital, Central South University, Changsha, Hunan, P.R. China.

**Keywords:** efficacy, glioma, lomustine, procarbazine, safety, vincristine

## Abstract

**Background:**

Systematic evaluation of the effectiveness and safety of combined procarbazine, lomustine, and vincristine for treating recurrent high-grade glioma.

**Methods:**

Electronic databases including PubMed, MEDLINE, EMBASE, Cochrane Library Central Register of Controlled Trials, WanFang, and China National Knowledge Infrastructure (CNKI) were used to search for studies related to the utilization of combined procarbazine, lomustine, and vincristine as a therapeutic method for recurrent high-grade glioma. Literature screening, extraction of data, and evaluation of high standard studies were conducted by 2 independent researchers. The robustness and strength of the effectiveness and safety of combined procarbazine, lomustine, and vincristine as a therapeutic methodology for recurrent high-grade glioma was assessed based on the odds ratio (OR), mean differences (MDs), and 95% confidence interval (CI). RevMan 5.3 software was used for carrying out the statistical analysis.

**Results:**

These results obtained in this study will be published in a peer-reviewed journal.

**Conclusion:**

Evidently, the conclusion of this study will provide an assessment on whether combined procarbazine, lomustine, and vincristine provides an effective and safe form of treatment for recurrent high-grade glioma.

**Systematic review registration number:**

INPLASY202080078.

## 1 Introduction

Gliomas refer to a type of prime brain tumors that form through supporting cells of the brain or spinal cord. Gliomas have been separated by the World Health Organization (WHO) as low-grade (I or II) and high-grade gliomas (grade III or IV). These classifications are based on characteristics reflecting aggressiveness and infiltration. There is a strong correlation between higher grade and poorer clinical outcomes. Grade III gliomas comprises of anaplastic astrocytoma (AA), anaplastic oligodendroglioma (AO), and anaplastic oligoastrocytoma (AOA or mixed glioma). Similarly, Grade IV gliomas are glioblastomas (GBM).^[1]^ Recently, the WHO updated the classification of central nervous system (CNS) tumors, which resulted in a major restructuring of diffuse gliomas, consequently, genetically defined entities were incorporated.^[2]^ With regards to treating recurrent high-grade glioma patients, currently, inhaled drugs, such as, procarbazine, has been instrumental for easing the symptoms and improving the life standard of a patient. Thus, it is essential to research for compelling and secure intervention for treating recurrent high-grade glioma.

Evidently, both empirical studies and clinical analysis have established that, combined procarbazine, lomustine, and vincristine has the capability to effectively treat the symptoms and improve the life standard of a patient. However, there has not been any systematic review aimed at evaluating the effectiveness and safety of combined procarbazine, lomustine, and vincristine for treating recurrent high-grade glioma. Consequently, the present study aims at conducting a systematic review to evaluate the effectiveness and safety of combined procarbazine, lomustine, and vincristine as a therapeutic means for treating recurrent high-grade glioma.

## 2 Materials and methods

This study has been registered at the International Platform of Registered Systematic Review and Meta-analysis Protocols (INPLASY, https://inplasy.com/). The registration DOI number of the current study is 10.37766/inplasy2020.8.0078. Furthermore, the reporting of this review protocol conforms with the Preferred Reporting Items for Systematic Review and Meta-Analyses (PRISMA) guidelines.^[3]^

### 2.1 Eligibility criteria

1. Types of studies
  Each study included was double-blind, randomized, and parallel-group, the studies were all aimed at evaluating combined procarbazine, lomustine, and vincristine as a treatment method for current high-grade glioma. Numerous other studies were excluded, such as, observational studies, non-randomized control studies, and case reports.
2. Types of participants
  The present study recruited adults (aged 18 years or older), the participants were treated previously for histologically confirmed grade III or IV glioma according to the criteria of the WHO during the initial diagnosis.
3. Types of interventions and comparisons
  The evaluations is inclusive of all variations of PCV chemotherapy in either arm, these evaluations have been carried out in terms of dosage, intensity, median number of cycles received, and duration of treatment. Additional salvage therapy encompasses corticosteroids, reirradiations with different dosages, and resurgeries either with or without BCNU chemotherapy-containing wafers within the tumor cavity (as long as it is similar in both arms). Moreover, the control arm was eligible to receive any of the following: placebo; best supportive care; an active intervention with second-line chemotherapy, or re-challenge with TMZ; anti-angiogenics (medications to inhibit the formation of new blood vessels within tumors); novel therapy, such as electrical stimulation; or combined medications that could comprise of 1 or 2 of procarbazine, lomustine, or vincristine.
4. Types of outcome measures
  (1) Primary outcomes
    The definition of the overall survival is time from randomization to death from any cause.
  (2) Secondary outcomes
    (i) The definition of progression-free survival (PFS) is the time from randomization to progression of disease.
    (ii) Either the European Organization for Research and Treatment of Cancer (EORTC) Core Quality of Life Questionnaire or Brain Cancer Module scale, or both the questionnaire and scale are used to measure quality of life.
    (iii) Participants who were experiencing chemotherapy toxicity were grouped. During the grading of toxicity, the process conformed with the Common Terminology Criteria for Adverse Events.

### 2.2 Search methods

1. Electronic searches
  Electronic databases such as, PubMed, MEDLINE, EMBASE, Cochrane Library Central Register of Controlled Trials, WanFang, and China National Knowledge Infrastructure (CNKI) were utilized to search for studies that were related to the utilization of combined procarbazine, lomustine, and vincristine for treating recurrent high-grade glioma. Each of the databases listed above will be searched from inception to the present, without any restrictions on the language and publication time.
2. Searching other resources
  In order to seek additional studies, Google Scholar, ClinicalTrials.gov (www.ClinicalTrials.gov), and reference lists from both the primary studies and review articles were searched.
3. Search strategy
  The search strategy includes the following keywords (“glioma” OR “recurrent high-grade glioma” OR “high-grade glioma”) AND (“procarbazine” OR “lomustine” OR “vincristine” OR “procarbazine, lomustine, and vincristine”) AND (“randomized clinical trial” OR “randomized controlled trial” OR “randomized” OR “RCT”).

### 2.3 Data collection and analysis

**Figure 1:**
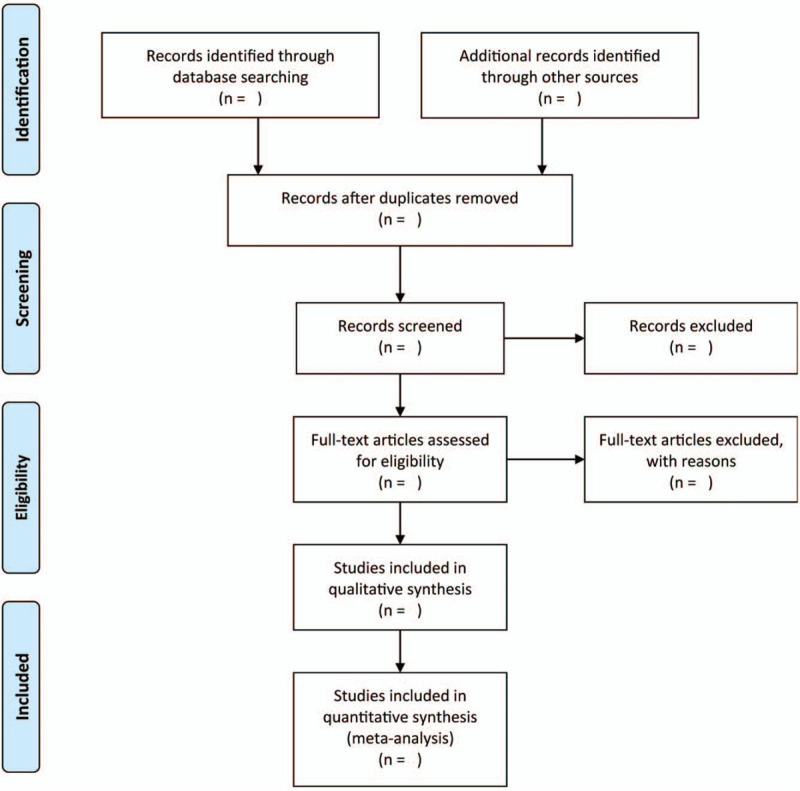
Flow diagram of the literature search.

1. Selection of studies
  Based on the eligibility criteria outlined previously, 2 independent scholars conducted literature selection. If a disagreement occurs, a discussion or the consultation of a third scholar shall be used to resolve the disagreement. The tiles and abstracts of the literature searched shall be identified to remove duplicates and studies that are not related to the theme. The process underlining the selection of studies is shown in the PRISMA flow chart (Fig. 1).
2. Data extraction
  For each included study, a minimum of 2 authors will excerpt related information independently, following this, the data will be imported into Excel tables. The information will consist of author, publication year, the criteria of diagnostic, eligibility criteria, details of treatment and control interventions, duration of intervention, and outcome indicators. In the event of any disagreement, a discussion or the consultation of a third scholar will be used to resolve the disagreement.
3. Assessment of study quality
  In accordance with the criteria outlined in the Cochrane Collaboration's tool,^[4]^ 2 authors conduct an independent evaluation of the quality of the studies included. In the event of any disagreement, a discussion or the consultation of a third scholar will be used to resolve the disagreement. The risk bias of each study included was assessed with the use of the following domains: selection bias, detection bias, reporting bias, performance bias, attrition bias, and other source of bias. Each potential source of bias will be graded according to 3 levels: “High risk,” “Low risk,” and “Unclear risk.”
4. Measures of treatment effect
  The dichotomous variables will be analyzed using RevMan 5.3 (Cochrane, London, UK), the analysis involves odds ratios (ORs) and continuous variables using the mean difference (MD) with its 95% confidence intervals (CIs).
5. Assessment of heterogeneity
  Standard Chi-squared statistic and *I*^2^ test will be used to detect the statistical heterogeneity across all included studies,^[5]^ where minor heterogeneity is implied by *I*^2^ < 50% or *P*-value > .1, and the data will be pooled with the use of the fixed-effects model.^[6]^ Meanwhile, considerable heterogeneity is implied by *I*^2^ > 50% or *P*-value < .1, and the data are pooled with the use of the random-effects model.^[7]^
6. Assessment of reporting biases
  Once the number of included studies exceeds 10, funnel plot and Egger's test will be adopted for evaluating the potential publication bias.^[8,9]^
7. Sensitivity analysis
  The stability and robustness of the process to find studies will be tested through a sensitivity analysis, this will be achieved by excluding studies identified with high-risk of bias or those containing unclear methodological data.

### 2.4 Ethics and dissemination

For the current systematic review, an ethical approval is not deemed necessary. The current study does not recruit any patients, nor does it collect any information from patients. This review will be disseminated via peer-reviewed journal.

## 3 Discussion

Admittedly, previously published studies have investigated combined procarbazine, lomustine, and vincristine as a means for treating recurrent high-grade glioma. However, the outcomes are largely controversial, and lack conclusiveness. Furthermore, there have not been any prior systematic reviews to evaluate the effectiveness and safety of combined procarbazine, lomustine, and vincristine for treating recurrent high-grade glioma. Consequently, the present systematic review aims to evaluate the effectiveness and safety of combined procarbazine, lomustine, and vincristine in the treatment of recurrent high-grade glioma. The results of this study could provide evidence which can be used by health-related professionals and clinicians to make clinical decisions that could enhance the treatment methods for recurrent high-grade glioma.

## Author contributions

**Conceptualization:** Yu-Gang Jiang, Ming Wang, Zhi-Gang Tan.

**Data curation:** Yang Cai, Yu-Gang Jiang, Ming Wang, Zhi-Gang Tan.

**Formal analysis:** Yang Cai, Ming Wang, Zhuo-Hang Jiang.

**Funding acquisition:** Zhuo-Hang Jiang.

**Investigation:** Yang Cai, Zhi-Gang Tan.

**Methodology:** Yang Cai.

**Resources:** Ming Wang, Zhi-Gang Tan.

**Software:** Yang Cai, Zhuo-Hang Jiang, Zhi-Gang Tan.

**Supervision:** Zhi-Gang Tan.

**Visualization:** Yang Cai.

**Writing – original draft:** Yang Cai, Zhi-Gang Tan.

**Writing – review & editing:** Yang Cai, Zhi-Gang Tan.

## Abbreviations

Abbreviations: CI = confidence interval, CNKI = China National Knowledge Infrastructure, CNS = central nervous system, GBM = glioblastomas, MD = mean differences, ORs = odds ratios, PFS = progression-free survival, WHO = World Health Organization.
